# Near-infrared spectra dataset of milk composition in transmittance mode

**DOI:** 10.1016/j.dib.2023.109767

**Published:** 2023-11-04

**Authors:** Jose A. Diaz-Olivares, Arnout van Nuenen, Martin J. Gote, Valeria Fonseca Díaz, Wouter Saeys, Ines Adriaens, Ben Aernouts

**Affiliations:** a Department of Biosystems, Division of Animal and Human Health Engineering, KU Leuven, Geel Campus, Kleinhoefstraat 4, 2440 Geel, Belgium; b Department of Biosystems, Division of Mechatronics Biostatistics and Sensors, KU Leuven, Kasteelpark Arenberg 30, 3001 Leuven, Belgium

**Keywords:** Near-infrared spectroscopy, Transmittance, Food quality control, Milk, Real-time prediction, Multivariate calibration, Calibration monitoring

## Abstract

Monitoring of milk composition can support several dimensions of dairy management such as identification of the health status of individual dairy cows and the safeguarding of dairy quality. The quantification of milk composition has been traditionally executed employing destructive chemical or laboratory Fourier-transform infrared (FTIR) spectroscopy analyses which can incur high costs and prolonged waiting times for continuous monitoring. Therefore, modern technology for milk composition quantification relies on non-destructive near-infrared (NIR) spectroscopy which is not invasive and can be performed on-farm, in real-time.

The current dataset contains NIR spectral measurements in transmittance mode in the wavelength range from 960 nm to 1690 nm of 1224 individual raw milk samples, collected on-farm over an eight-week span in 2017, at the experimental dairy farm of the province of Antwerp, ‘Hooibeekhoeve’ (Geel, Belgium). For these spectral measurements, laboratory reference values corresponding to the three main components of raw milk (fat, protein and lactose), urea and somatic cell count (SCC) are included.

This data has been used to build multivariate calibration models to predict the three milk compounds, as well as develop strategies to monitor the prediction performance of the calibration models.

Specifications Table

**Table:** 

Subject	Near-infrared spectroscopy
Specific subject area	Chemometrics
Type of data	Tables Figures Spectroscopic data
How the data were acquired	For all milkings performed by an automatic milking system (AMS), an on-farm long-wave near-infrared (LW-NIR, 960–1690 nm) analyzer measured the transmittance spectra of a representative raw milk sample. These measurements were acquired using a 256-pixel cooled InGaAs diode array NIR spectrometer (1.7-256 Plane Grating Spectrometer, Carl Zeiss, Jena, Germany) with a resolution of 2.86 nm per pixel, fixing an integration time of 100 ms and averaging 100 repeated measures. For the same sample, white and dark reference spectra were also acquired, and laboratory reference values for fat, protein, lactose, urea and somatic cell count (SCC) were obtained following ISO 9622 and ISO 13366-2:2006.
Data format	Raw Normalized
Description of data collection	The dataset is a .csv file with 1224 NIR transmittance measurements of raw milk, which with its corresponding white and dark reference it is normalized with the following: $\frac{\text{sample}\;\text{raw}\;\text{spectrum}-\text{dark}\;\text{spectrum}\;}{\text{white}\;\text{spectrum}-\text{dark}\;\text{spectrum}}$. The data set also contains the time in which all measurements and milkings were performed. These spectral measurements have laboratory reference values for fat, protein, lactose, urea and somatic cell count (SCC).
Data source location	Raw milk samples were extracted and measured at the experimental farm of the province of Antwerp, Hooibeekhoeve (Hooibeeksedijk 1, 2440 Geel, Belgium). Laboratory references of the raw milk samples were performed by the milk control center MCC-Vlaanderen (Hagenbroeksesteenweg 167, 2500 Lier, Belgium). Experimental design, NIR analyzer development and spectral data treatment were performed by the Livestock Technology Laboratory of KU Leuven (Kleinhoefstraat 4, 2440 Geel, Belgium).
Data accessibility	Data was deposited at a publicly available Zenodo repository. https://doi.org/10.5281/zenodo.8263430
Related research article	J.A. Diaz-Olivares, I. Adriaens, E. Stevens, W. Saeys, and B. Aernouts, Online milk composition analysis with an on-farm near-infrared sensor. Comput. Electron. Agric. 178 (2020) 105734. https://doi.org/10.1016/j.compag.2020.105734

## 1 Value of the Data

• Extensive datasets with multiple domains and time dimensions for spectroscopy are necessary for methodological innovations on how to build more robust calibration models and how to maintain them in time.
• This dataset contains a large number of samples with raw information which enables the investigation of more robust approaches to filter noise from signals and validate their stability.
• This dataset contains white and dark spectral reference measurements for each milk sample analyzed. These data allow to investigate the optimal frequency of taking into account a new set of spectral reference measurements to achieve maximum prediction performance.
• Availability of five chemical compounds of milk composition. Some of those compounds are harder to predict than others, enabling the exploration of many chemometrics and machine learning models to find better models.
• Researchers and industrial practitioners involved in multivariate calibration will be able to benchmark their accuracy with published studies that have used this data.
• The dataset provides an opportunity for chemometricians to analyze the variability and distributions of the chemical compounds in milk, enabling the development of more sophisticated models for predicting and monitoring changes in milk composition.

## 2 Objective

Quantify milk composition based on non-destructive spectroscopy in transmittance mode. This is achieved by building multivariate calibration models that predict the chemical parameters based on spectral variables. The quantification of milk composition has been applied in the development of online monitoring systems in dairy farms [1], methodological research about optimal sample sizes for calibration models [2], and proposals for calibration monitoring methods [3].

## 3 Data Description

**Fig. 1:**
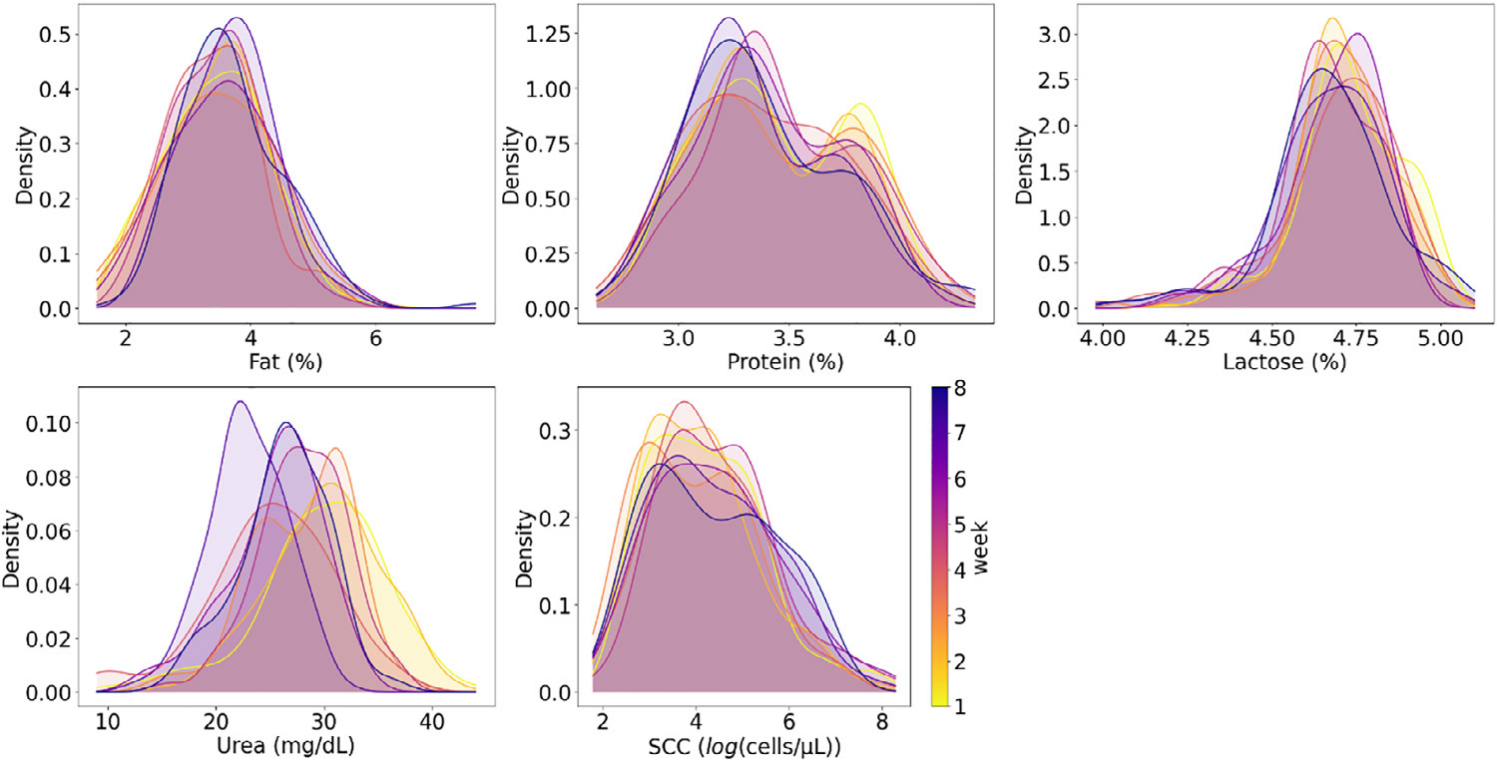
Distribution of milk compounds per week.

**Fig. 2:**
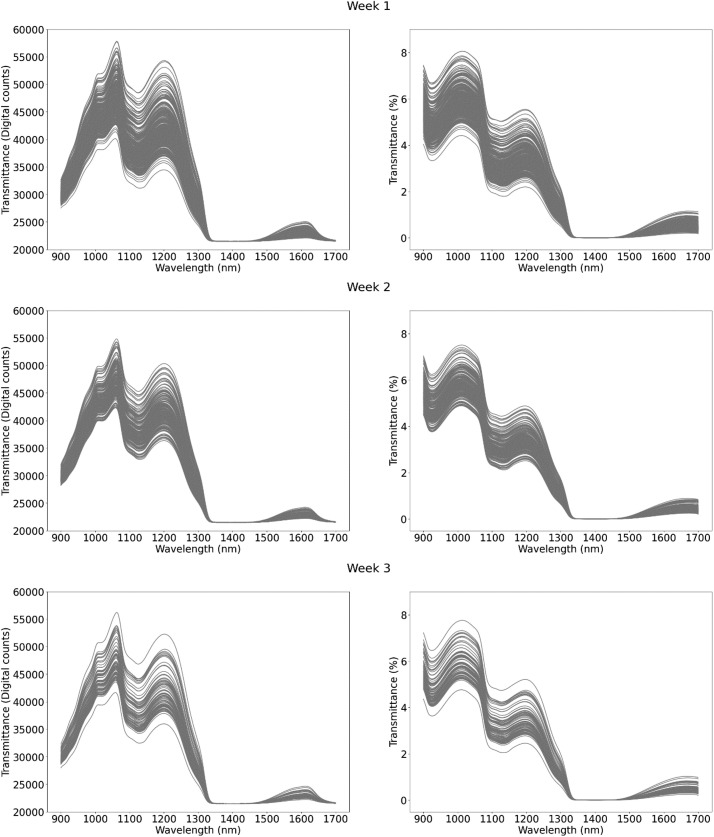

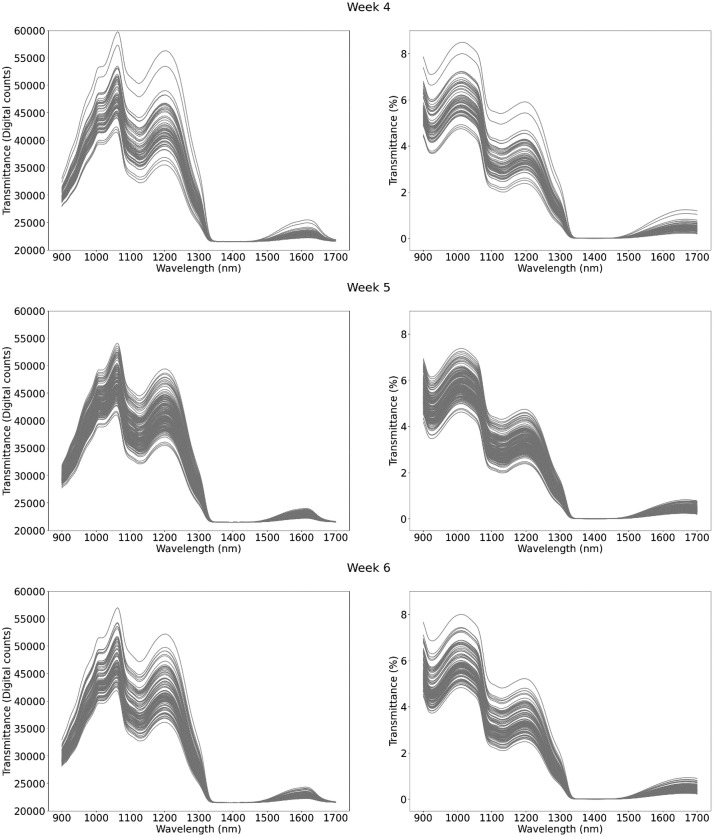

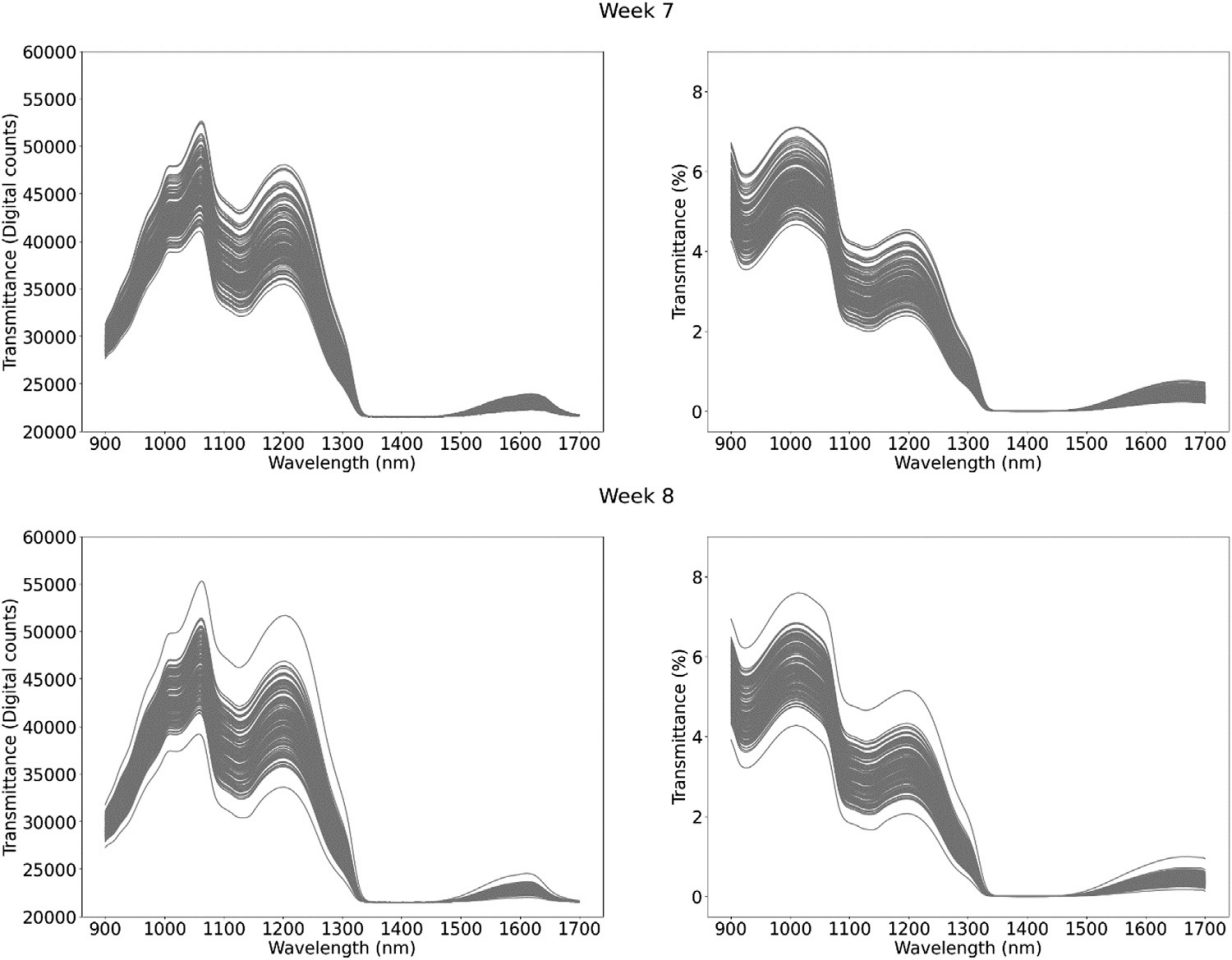
Weekly spectral measurements in transmittance mode. The plots on the left display the raw transmitted spectra measured in digital counts. The plots on the right show the normalized transmittance spectra, calculated according to the formula: $\frac{\mathrm{sample}\;\mathrm{raw}\;\mathrm{spectrum}-\mathrm{dark}\;\mathrm{spectrum}\;}{\mathrm{white}\;\mathrm{spectrum}-\mathrm{dark}\;\mathrm{spectrum}}$**.**

This dataset contains 1224 NIR spectral measurements of raw milk samples, captured over the course of eight weeks at the experimental farm of the province of Antwerp “Hooibeekhoeve” (Geel, Belgium). The spectral measurements are available in transmittance mode in the wavelength range of 960–1690 nm, with a resolution of 2.86 nm/pixel and a total of 256 wavelengths. All transmittance spectral measurements include: (1) a measurement of the correspondent raw milk sample; (2) a measurement of a white spectral reference; and (3) a measurement of a dark spectral reference. All measurements had an integration time of 100 ms and averaged 100 measurements. Additionally, they have corresponding laboratory reference values for the three main components of raw milk (fat, protein and lactose), urea and somatic cell count (SCC). Each entry corresponds to a single milk sample, with all milk samples being taken from 41 cows. The data is provided as a .csv file, with each variable listed and described in Table 1**.** A summary of the reference values for the five milk compounds (Table 2 and Fig. 1) and the spectral measurements (Fig. 2: Weekly spectral measurements in transmittance mode. The plots on the left display the raw transmitted spectra measured in digital counts. The plots on the right show the normalized transmittance spectra, calculated according to the formula: ) are presented for each measurement week.

**Table 1:** Fields included in the data file with description and units.

Table	Description	Units
Cow_ID	Identification number of each measured cow	-
Fat	Milk fat content	% w/w
Prot	Milk protein content	% w/w
Lact	Milk lactose content	% w/w
SCC	Milk somatic cell count	cells/µL
Urea	Milk urea content	mg/dL
Milk_Yield	Total milk yield	Liters
Milk_Interv	Time difference since the last milking for the cow being currently milked	Seconds
SET	Week (1,2,3,4,5,6,7,8)	Week
Time_Dark	Timestamp of white spectral reference measurement	Seconds^a^
Time_Milk	Timestamp of end of milking process	Seconds^a^
Time_PrevMilk	Timestamp of the last milking for the cow being currently milked	Seconds^a^
Time_Sample	Timestamp of sample spectral measurement	Seconds^a^
Time_White	Timestamp of white spectral reference measurement	Seconds^a^
Trans_Dark^b^	Dark spectral reference measurement	Digital counts^c^
Trans_Sample^b^	Sample spectral transmittance measurement	Digital counts^c^
Trans_Tot^b^	Normalized sample transmittance spectra	%
Trans_White^b^	White spectral reference measurement	Digital counts^c^

a Indication that time for this variable is measured in seconds from 01/01/1970 at 00:00:00 (Unix time); other time measurements are relative.

b Spectral measurements comprising 256 variables. These variables correspond to the 256 pixels, which are distributed across a wavelength range of 960 nm to 1690 nm, at a resolution of 2.86 nm per pixel.

c Indication that this is a 16-bit value that ranges from 0 to 65535.

**Table 2:** Summary of milk composition.

Week	N	Fat		Protein		Lactose		Urea		SCC	
		Mean	Std	Mean	Std	Mean	Std	Mean	Std	Mean	Std
1	348	3.45	0.85	3.45	0.34	4.74	0.15	29.92	5.83	172.90	400.54
2	190	3.45	0.80	3.42	0.33	4.72	0.14	30.08	5.05	132.25	249.71
3	79	3.50	0.90	3.44	0.36	4.70	0.16	28.04	4.48	139.91	300.26
4	60	3.34	0.76	3.40	0.34	4.70	0.17	25.10	5.66	130.03	204.82
5	167	3.47	0.72	3.47	0.33	4.67	0.15	28.07	4.04	227.13	503.50
6	96	3.58	0.86	3.41	0.32	4.69	0.14	25.50	4.30	237.30	500.77
7	168	3.66	0.72	3.36	0.32	4.67	0.17	22.99	3.60	191.02	362.29
8	162	3.72	0.83	3.39	0.34	4.67	0.18	26.30	4.07	187.75	255.78

### 3.1 Experimental design, materials and methods

An NIR spectroscopic analyzer was integrated on-farm to predict the milk composition of cow milkings performed by a VMS™ (DeLaval, Sweden) automatic milking system (AMS). For each individual milking, a minimum of 150 mL of raw milk, representative of the milk produced during that milking, was collected by the analyzer which was bypassing the Herd Navigator™ (HNS Supra+, DeLaval) sampler’s backflow, integrated into the AMS. An additional sample fraction (±30 mL), also representative of the milk produced during that milking, was dispatched to a VMX™ sampling device (DeLaval) to be collected in plastic sample tubes for laboratory milk analysis following milk recording programs [4].

The on-farm analyzer was composed of several elements housed within a compact, temperature-regulated steel casing (PowerCool, Laird, Liberec, Czech Republic). These elements included: (1) an NIR spectrometer (1.7-256 Plane Grating Spectrometer, Carl Zeiss, Jena, Germany) equipped with a cooled 256-pixel InGaAs (indium-gallium-arsenide) diode array with a resolution of 2.86 nm per pixel in the 960–1690 nm wavelength range; (2) a 20 Watt integrated halogen light source (998079-14, Welch Allyn, New York, USA); (3) a mechatronic support system, which utilized a peristaltic pump (EZ OEM Pump, Verderflex, the Netherlands) to regulate the flow of milk from the bypass to the cuvette for spectral analysis and consequent evacuation of the milk after the analysis; (4) a computer that executed the spectral acquisition and control software utilizing LabView 2012 (National Instruments, Austin, USA); and (5) an optical measurement unit.

The latter consisted of a custom-made translation stage, equipped with an automated stepper motor (L4118L1804, Nanotec, Munich, Germany) and a filter slide (FS40, OWIS, Frankfurt, Germany). The filter slide held a flow-through borosilicate cuvette with a 2 mm internal thickness and 26 mm diameter (1.2 mL volume), a 2 mm thick Spectralon plate (Labsphere, North Sutton, USA) working as a white spectral reference and a 1% Acktar Black coated plate (Acktar, Hohenaspe, Germany) as a dark spectral reference. The translation stage facilitated the alternating measurement of spectral references before measuring the raw milk sample in the cuvette. The transmitted light was collected by a converging-type lens and directed to the spectrometer via an optical fiber with low OH and a 600 µm core diameter. The translation stage was designed to move perpendicularly to the lens-halogen axis, with the lens aligned to the halogen light source.

The NIR spectrometer recorded transmittance spectra for each raw milk sample placed in the cuvette, in the wavelength range from 960 to 1690 nm. To enhance the signal-to-noise ratio, 100 repeated measurements of the milk sample were gathered at 100 ms integration time and then combined into a single average spectrum per sample. Furthermore, the white and dark spectral reference measurements were captured using the same parameters, right before measuring the milk sample spectrum, simultaneous to the process of filling the cuvette with the milk sample.

The measurements of both the white and dark spectral references were recorded to characterize alterations in the intensity spectrum of the light source and the spectral sensitivity of the spectrometer. These alterations can be the result of stray light, fluctuations in environmental temperature, or wear of the lamp filament [5]. The sample spectra were normalized using a pair of white and dark spectral reference measurements according to $\frac{\text{sample}\;\text{raw}\;\text{spectrum}-\text{dark}\;\text{spectrum}\;}{\text{white}\;\text{spectrum}-\text{dark}\;\text{spectrum}}$. This correction step is crucial to correct for alterations in light source intensity and spectrometer sensitivity. However, taking into account new spectral reference measurements for every new milk sample measurement can also introduce more noise than it removes, especially if alterations in light source intensity and spectrometer sensitivity between two subsequent spectral reference measurements are much smaller than the (random) noise or difference between these subsequent measurements. Thus, if consequent spectral references are obtained at a frequency that exceeds an optimum limit, additional random noise, commonly found in spectral measurements, will be introduced unnecessarily. To maintain the accuracy of the milk composition predictions, the intervals at which white and dark spectral reference measurements should be obtained were evaluated. It was determined that when performing the normalization of the spectra, the value of the spectral reference pair should only be updated immediately at the beginning of each spectral recording and sample acquisition session, right after the warm-up and stabilization of the light source to a nominal point, and then every 0.5 h of measurements thereafter [1].

For a span of eight weeks, the analyzer measured raw milk samples collected from 41 Holstein cows at the “Hooibeekhoeve” dairy farm in the province of Antwerp (Geel, Belgium). The cows, at an average lactation stage of 168 ± 84 days in milk and parity of 2.0 ± 1.1, were milked 2.6 times daily on average. However, spectral analysis was only carried out when a sample was captured for reference analysis as part of an unrelated feeding trial. The duration of milk sample collection ranged from 21 to 85 h per week, averaging 158 samples collected each week. The raw milk samples collected for laboratory analysis were treated with 0.3 mg/mL of bronopol and underwent analysis at the Milk Control Center (MCC Vlaanderen, Lier, Belgium) within no more than three days following sample collection. The laboratory reference values for fat, protein, lactose and urea for each sample were determined in accordance with ISO 9622 [6] and with ISO 13366-2 [7] for SCC. For these laboratory analyses, the CombiFoss™ FT+ (Foss A/S, Hillerød, Denmark) instrument was utilized, with an accuracy of <1.0% coefficient of variation (CV) for fat, under 0.9% CV for protein and lactose, a standard deviation of < 3 mg/dl for urea and a <10% relative mean difference from direct microscopic SCC.

In total, 1270 individual milkings with their corresponding milk samples and spectral measurements were captured during the experimental period, corresponding to 22% of the total milkings (*n* = 5969) in that period of eight weeks. Forty-six of these samples have no compositional laboratory reference as a consequence of a failure of the VMX™ sampling device to acquire sufficient milk to fill the sample tubes. Therefore, 1224 raw milk samples for which both laboratory reference analyses and transmission spectra are available.

## Ethics Statement

The milk samples used for this study were collected without interfering with the normal management of the cows and the daily management of the farm. Although animals were needed to produce the milk for this study, it does not classify as an animal experiment.

## CRediT authorship contribution statement

**Jose A. Diaz-Olivares:** Writing – original draft, Investigation, Data curation, Methodology, Writing – review & editing. **Arnout van Nuenen:** Data curation, Software, Writing – review & editing. **Martin J. Gote:** Data curation, Software, Writing – review & editing. **Valeria Fonseca Díaz:** Writing – original draft, Visualization, Software. **Wouter Saeys:** Funding acquisition, Supervision, Writing – review & editing. **Ines Adriaens:** Funding acquisition, Supervision, Writing – review & editing. **Ben Aernouts:** Conceptualization, Methodology, Investigation, Data curation, Funding acquisition, Project administration, Resources, Supervision, Writing – review & editing.

## Data Availability

NIR spectra dataset of milk composition in transmitance mode (Original data) (Zenodo)
